# FastEmbed: Predicting vulnerability exploitation possibility based on ensemble machine learning algorithm

**DOI:** 10.1371/journal.pone.0228439

**Published:** 2020-02-06

**Authors:** Yong Fang, Yongcheng Liu, Cheng Huang, Liang Liu

**Affiliations:** College of Cybersecurity Sichuan University, Chengdu, Sichuan, P.R.China; Vietnam National University, VIET NAM

## Abstract

In recent years, the number of vulnerabilities discovered and publicly disclosed has shown a sharp upward trend. However, the value of exploitation of vulnerabilities varies for attackers, considering that only a small fraction of vulnerabilities are exploited. Therefore, the realization of quick exclusion of the non-exploitable vulnerabilities and optimal patch prioritization on limited resources has become imperative for organizations. Recent works using machine learning techniques predict exploited vulnerabilities by extracting features from open-source intelligence (OSINT). However, in the face of explosive growth of vulnerability information, there is room for improvement in the application of past methods to multiple threat intelligence. A more general method is needed to deal with various threat intelligence sources. Moreover, in previous methods, traditional text processing methods were used to deal with vulnerability related descriptions, which only grasped the static statistical characteristics but ignored the context and the meaning of the words of the text. To address these challenges, we propose an exploit prediction model, which is based on a combination of fastText and LightGBM algorithm and called fastEmbed. We replicate key portions of the state-of-the-art work of exploit prediction and use them as benchmark models. Our model outperforms the baseline model whether in terms of the generalization ability or the prediction ability without temporal intermixing with an average overall improvement of 6.283% by learning the embedding of vulnerability-related text on extremely imbalanced data sets. Besides, in terms of predicting the exploits in the wild, our model also outperforms the baseline model with an F1 measure of 0.586 on the minority class (33.577% improvement over the work using features from darkweb/deepweb). The results demonstrate that the model can improve the ability to describe the exploitability of vulnerabilities and predict exploits in the wild effectively.

## Introduction

Information security vulnerabilities pose a significant and increasing threat to national security and economic well-being. A vulnerability is a weakness of a software product that can be exploited by an attacker to compromise the confidentiality, integrity, or availability of the system hosting that product and cause harm [1] and may produce incalculable severe results. For example, on May 12, 2017, a trojan malware named WannaCry exploits a known vulnerability in the Windows operating system, infecting more than 230,000 computers in endless core sectors across more than 150 countries [2]. As a large number of vulnerabilities especially significant severe vulnerabilities [3] are disclosed by the National Institute of Standards and Technology (NIST) every year on an upward trend [4], classifying and avoiding risks as much as possible have become urgent problems for enterprises and security experts. For example, when the vulnerabilities related to WannaCry is disclosed in NVD on March 16, 2017, if there is a method that can analyze the relevant information mentioned online of the vulnerabilities and judge the exploitability of the vulnerability, under the condition that the method is effective, the losses of enterprises and individuals will be greatly reduced after 57 days.

The relationship between risk and precaution is becoming extremely tense. On the one hand, the number of vulnerabilities disclosed in National Vulnerability Database (NVD) is increasing almost every year–NVD released 16,555 and 9,526 vulnerabilities in 2018 and the first eight months of 2019 respectively, showing significant growth compared with previous years such as 6,484 vulnerabilities in 2015. After a vulnerability is disclosed, the possibility of its exploitation increases dramatically [5]. At the same time, the increasing number of vulnerabilities incites cyberattackers to disclose security exploits by taking advantage of the Internet’s sharing capabilities [6], bringing more severe risks to the unpatched systems. On the other hand, less than 3% of published vulnerabilities are found to be exploited in the wild [7–12]. The vast majority of vulnerabilities are never exploited by attackers while the proportion of exploitation decreases over time [10, 12]. Furthermore, some existing methods such as Common Vulnerability Scoring System (CVSS) score [13, 14] are often biased against facts when applied to patch prioritization [9].

The previous work used machine learning technology to describe the relationship between vulnerabilities and Proof-of-Concept (PoC) exploits [8, 15, 16, 17] or to predict exploits in the wild through vulnerabilities mentioned online [9, 18, 19], making some progress by using diversified threat intelligence information including vulnerability database and darkweb/deepweb and attracting the attention of industry [20]. However, some problems exist in the past method. First, in terms of intelligence sources, Twitter [11] has proved to be not robust [15]. The data in darkweb/deepweb shows excellent prediction performance [21] but is narrow in coverage. The vulnerability database is the most authoritative and comprehensive source of vulnerability data. Nevertheless, the vulnerability database suffers performance losses caused by the delay of the population of CVSS metrics and the rapid shortening of vulnerability exploitation time [22]. Second, when dealing with text associated to vulnerabilities, traditional text processing techniques represent statistical features of words, but fail to capture their context and identify the meaning of rare words and subword such as the relation between “virus” and “anti-virus”. The last but not the least, no matter whether the vulnerability data is labeled with PoC exploits or exploits in the wild, the prediction model is facing the dilemma of a sharp increase in data imbalance.

To address these challenges, we describe an integrated model, which uses a neural network model [23, 24] to embed vulnerability-related text to derive the inherent meaning of the text. Then the classification results derived by the text embedding model or text embedding are combined with other features to predict the exploitation of vulnerabilities by applying LightGBM algorithm [25] on unbalanced data. Specifically, databases such as SecurityFocus that have more advantages in the efficiency of release time and vulnerability summary can be adopted as supplements to NVD database. Then, we employ the above model to carry out experiments on vulnerability data labeled with PoC exploits and exploits in the wild respectively. Specially, we put emphasis on the data labeled with exploits due to the reason that the majority of all the exploited vulnerabilities have available exploits within a week from the time of disclosure [26, 27] and the ground truth of exploits in the wild is biased towards existing in software products from specific vendors such as Microsoft and Adobe [11].

The model we proposed is effective as it captures the semantics and morphology of words and performs well on unbalanced data sets. We reproduced key portions of the representative work and used them as a benchmark model. Evaluations show that our model outperforms the baseline model whether in terms of the generalization ability or the prediction ability without temporal intermixing. Specifically, the major contributions of our paper include:

- We propose an integrated model named fastEmbed to predict whether a vulnerability published in vulnerability databases will have PoC exploits or be exploited in the wild. Evaluations demonstrate that fastEmbed outperforms the baseline model with an average overall improvement of 6.283% in predicting PoC exploits and an average overall improvement of 33.577% in terms of predicting exploits in the wild.
- FastEmbed can captures the semantics and morphology of words in the vulnerability-related text, which is proved to be one of the most important features of the model in this paper.
- FastEmbed predicts the release of PoCs a median of 1 day ahead and detects exploited vulnerabilities in security blogs more effectively than previous models.

The remainder of this paper is organized as follows. We give a review of related work in the field of predicting exploits using machine learning in Section 2. In Section 3 we describe our selection of data sources and introduce the proposed integrated model fastEmbed in detail. Section 4 describes our performance metrics, experimental setup, and comparative analysis of the experiment results. Finally, the conclusions and prospect of exploits prediction are laid out in Section 5.

## Related work

Assessing and prioritizing the threats of cybersecurity have gained ever-increasing attention in recent years [28–31]. Especially, several works have been devoted to measuring the risk of software vulnerabilities and predicting the future threat caused by software vulnerabilities, thus realizing vulnerability patching prioritization. Zhang et al. [32] attempted to predict the time of the next vulnerability for a given software application by using Common Vulnerability Scoring System (CVSS) and the Common Platform Enumeration (CPE) designed by the National Institute of Standards and Technology (NIST) as features rather than the NVD description. CVSS calculates the vulnerability risk index through different characteristics, which measures the availability and exploitability of a vulnerability [13, 14]. However, this indicator is not effective as it marks most vulnerabilities that do not have exploitation value for hackers as exploitable [9], which is equivalent to randomly selecting vulnerabilities to patch [33].

Recent works extract features from publicly available online vulnerability information and combine them with machine learning techniques to predict whether a given vulnerability will be exploited or exploited in the wild, with CVSS as one of the features of training. Among them, “exploited” means the availability of proof-of-concept exploit, and “exploited in the wild” indicates actual exploits that were used by hacker in the wild. Bozorgi et al. [16] leveraged the Open Source Vulnerability Database (OSVDB) and the NVD to train a support vector machine (SVM) classifier [34] as to predict whether a corresponding PoC will be developed for each vulnerability. They achieved excellent prediction results with an accuracy of 90% using a resampled and balanced data set with most vulnerabilities are marked as exploited in OSVDB. Bozorgi et al. extracted sufficient features from the vulnerability databases and achieved higher accuracy than CVSS vulnerability scoring system in vulnerability exploitation classification by means of machine learning method. With the increasing number of vulnerabilities and the closure of OSVDB database, the PoCs that references to CVE-IDs in Exploit Database (EDB) was used by Edkrantz and Said [8] to mark the exploitability of vulnerabilities and 27% of vulnerabilities are labeled as exploited. Their work gets a prediction accuracy of 83% for binary classification and shows that the vulnerability descriptions, vendor products and external references, are the most important features to consider. At the same time, they proved that the quality of the data set largely determines the prediction effect of the model.

Due to the inefficiency of NVD in reporting vulnerabilities, some previous works leveraged vulnerability-related contexts in social networks such as Twitter [11] and darkweb/deepweb forums [35] to build an early prediction model. Based on the work of Bozorgi [16], Sabottke et al. [11] use a linear SVM classifier to predict whether the tweets related to CVE vulnerabilities on Twitter will bring about the exploitation of vulnerabilities. In addition to predicting PoC exploits, they also predicted exploits that occurred in the real world based on Symantec’s anti-virus attack signatures [36] and Intrusion Detection Systems’ (IDS) attack signatures [37]. Only 1.3% of the vulnerabilities in Sabottke’s dataset are exploited in the wild, and 6.2% exploited, showing a sharp decline compared with previous work, which is consistent with our work. Sabottke et al. realize the early exploit threat prediction using social media for the first time and can predict exploits a median of 2 days ahead of existing data sets. Nazgol et al. [20] address the challenge that the past methods in vulnerability exploit prediction only captured the statistical features of words and ignored the context of words. They use a neural network model that learned the similarity of related posts on exploited vulnerabilities in semanteme and context by embedding posts from darkweb/deepweb into vector space to improve predictions of exploits in the wild. By combining the embeddings of posts with other features of the vulnerability such as CVSS score, their model predicted 1886 vulnerabilities with distinct CVEs in darkweb/deepweb and reached F1-score of 0.74. However, the representations of posts learned by their model ignore the morphology of words especially for rare words and lost some internal information of words.

The work carried out by Bullough et al. [15] replicates representative experiments in the past and points out that some unrealistic methods of past experiments have exaggerated the predictive performance of the models. Bias on the predictive ability for future vulnerabilities caused by balancing data sets and concept drift [38, 39] caused by temporal intermixing of training and test sets are common in past experiments. In this paper, we will replicate previous representative works and carry out empirical analysis on the real performance of our model.

## Methodology

**Fig 1:**
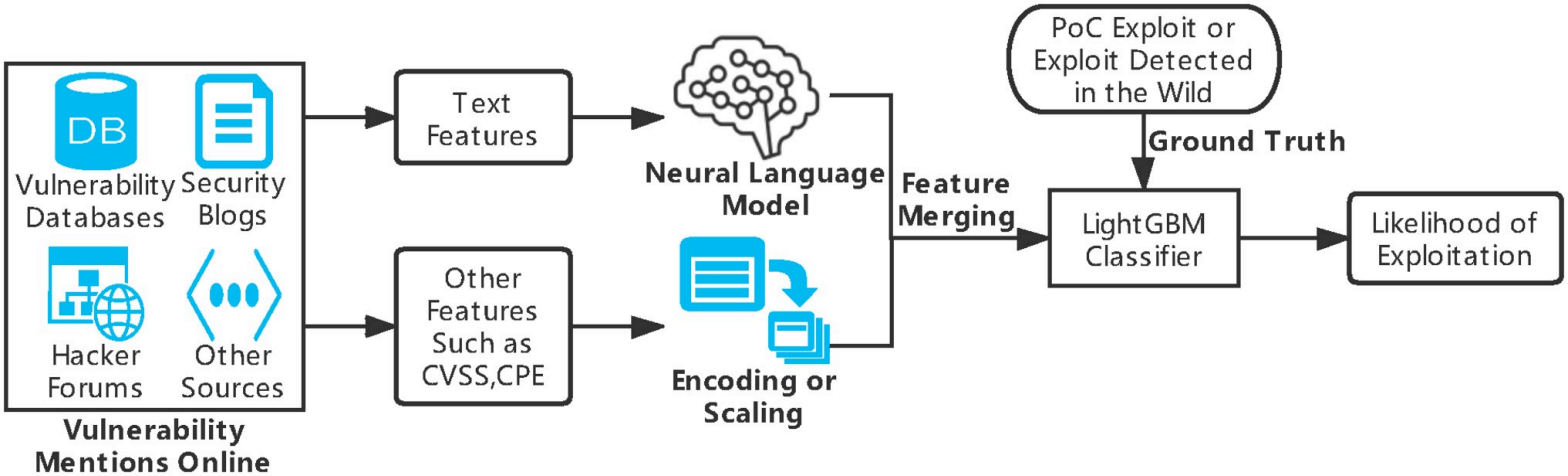
Overview of fastEmbed.

Our vulnerability prediction model consists of two components: feature merging and vulnerability classification. In the feature merging part, the input features of the model are divided into two categories, namely text features and other inherent features of vulnerabilities such as CVSS. In terms of text features, through lemmatization, removal of stop words, embedding of the neural language model, we get a more accurate embedded representation of vulnerability-related text. Judging from the output results of the text feature training model, through the training of neural network model, we can get the embedded representation of the vulnerability-related text or the classification result of whether the vulnerability is exploited from the vulnerability-related text. In the final step of feature merging, the result of text feature model classification or embedded representation of text features is combined with other robust features as the last feature. Then we use a two-class machine learning model to predict the possibility of vulnerabilities being exploited by hackers and to prioritize vulnerabilities to be remedied based on the above fusion of text features and inherent features of vulnerabilities, in which positive samples indicate that the vulnerabilities are exploitable, while negative samples indicate the opposite. Fig 1 gives an overview of the proposed exploit prediction approach.

### Data collection

The features fed into fastEmbed are extracted from open source intelligence data such as NVD. The label of the prediction model is determined by indicating whether there exists corresponding PoC or whether the exploitation of a vulnerability is detected. We chose the most representative and effective data proved by previous work to carry out the experiments. In order to reflect the timeliness of the model, we crawled the data sources from 2013 to 2018. Details of the data set are as follows.

#### NVD

**Fig 2:**
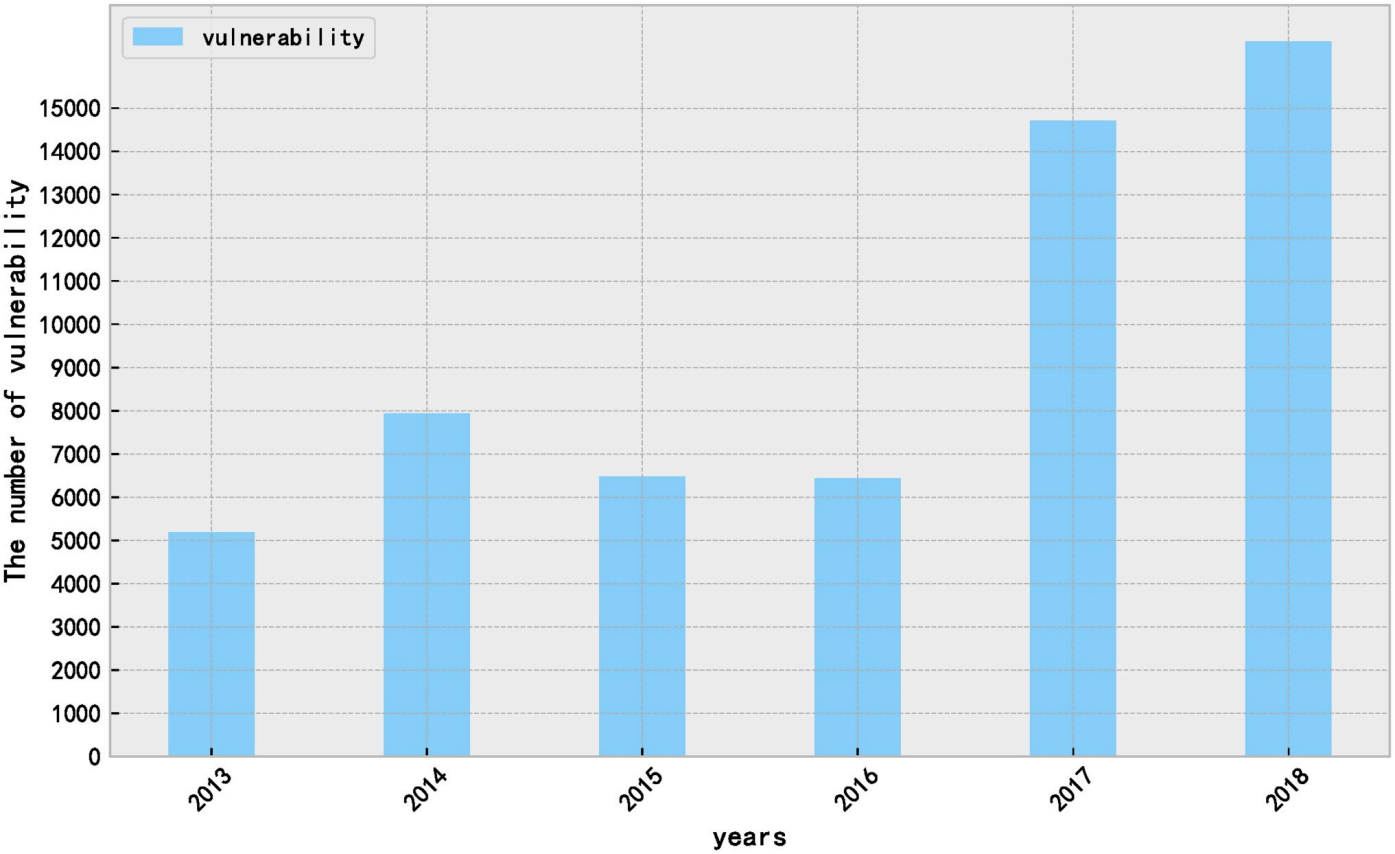
Vulnerability disclosures added to the National Vulnerability Database (NVD) by year.

The National Vulnerability Database is a repository of standards-based vulnerability management data. Vulnerabilities in the NVD database are indexed by the Common Vulnerabilities and Exposures Identifier (CVE-ID) [40]. Every year the NVD database provides the vulnerability data feeds consist of CVE-IDs allocated for that year in JSON format. We wrote python scripts to crawl the data from 2013 to 2018 from NVD and parse its fields. A dataset comprised of 60,784 vulnerabilities is formed. Fig 2 shows the distribution of the number of disclosures of vulnerabilities over time. Each entry includes a descriptive text summary for the vulnerability, CVSS scores and related indicators, information about affected products and vendors, the category of vulnerability based on the Common Weakness Enumeration (CWE) system, and reference URLs.

#### SecurityFocus

**Fig 3:**
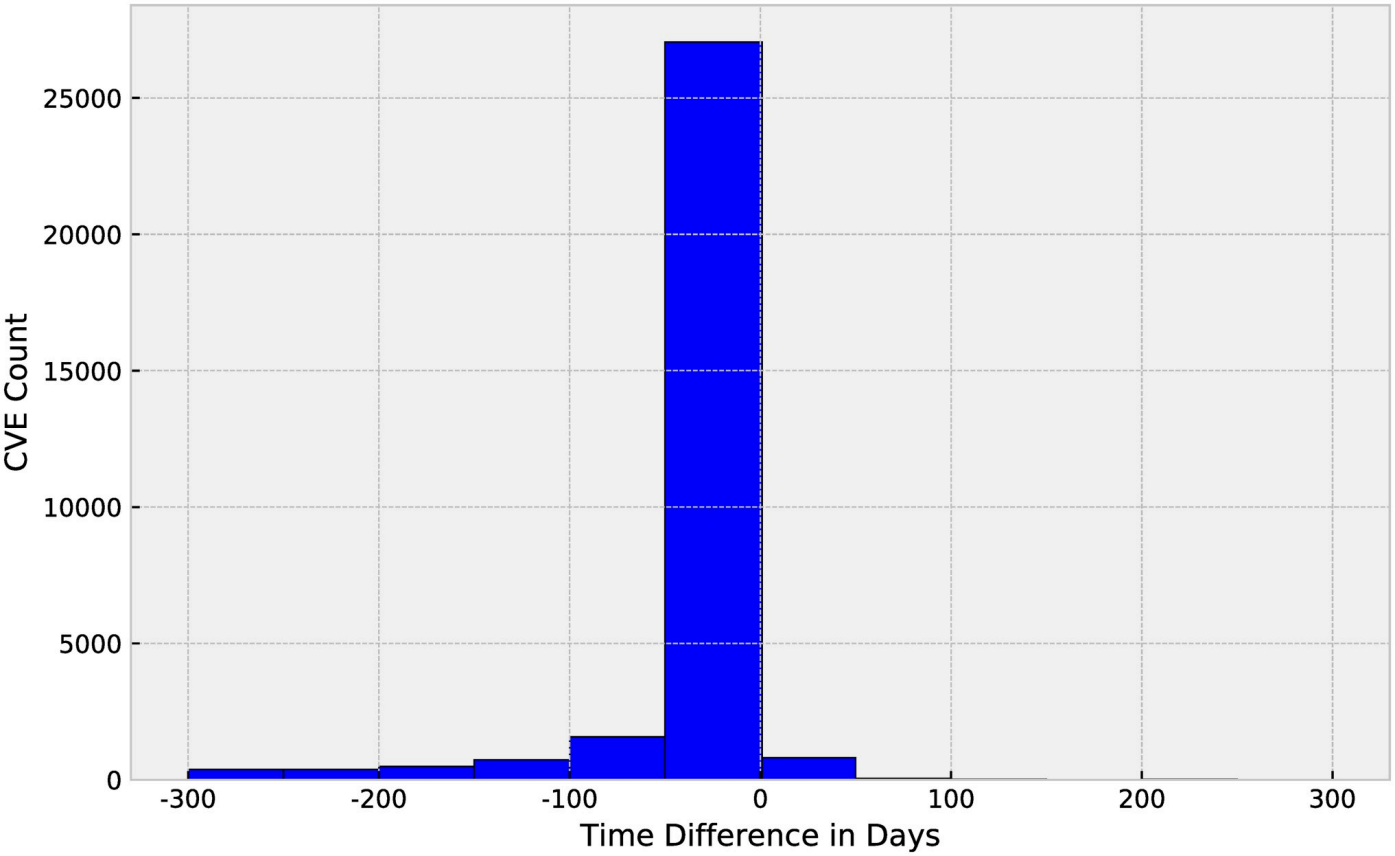
Distribution of the difference in days between vulnerability disclosure of NVD and SecurityFocus.

The SecurityFocus is one of the most prominent and well-respected vulnerability databases [41]. For security researchers, the NVD database provided the most complete and authoritative vulnerabilities in the world. Why do we choose the second largest database to carry out model verification [6, 41]? We crawled vulnerabilities on SecurityFocus and NVD from 2013 to 2018. After analyzing the data, we confirmed that SecurityFocus covers 54% of vulnerabilities on NVD and only 2.86% of the vulnerabilities with CVE references in SecurityFocus lag behind the NVD database in publishing time. Fig 3 shows a histogram of the difference in days between vulnerability disclosure in the NVD database and SecurityFocus. Moreover, eight graduate students in our laboratory conducted a manual review of some crawled vulnerabilities mainly in terms of standardization and semantic clarity of vulnerability description. Through our manual inspection of the vulnerabilities on NVD and SecurityFocus, vulnerability description of SecurityFocus has more reference significance for the prediction task in many cases. The two summaries below illustrate these differences.

- “Mirion Technologies multiple Telemetry enabled devices are prone to multiple security-bypass vulnerabilities. Successfully exploiting these issues may allow an attacker to bypass certain security restrictions and perform unauthorized actions. This may aid in further attacks.”
- “A Use of Hard-Coded Cryptographic Key issue was discovered in Mirion Technologies DMC 3000 Transmitter Module, iPam Transmitter f/DMC 2000, RDS-31 iTX and variants (including RSD31-AM Package), DRM-1/2 and variants (including Solar PWR Package)…”

The first summary comes from SecurityFocus (BID: 100001) and the second from NVD (CVE-ID: CVE-2017-9649). Given the words in the two summaries, the first summary seems to describe the impact and exploitability of the vulnerability more specifically. Based on the above facts, we use data on SecurityFocus and NVD to carry out experiments respectively for comparison. 29,867 vulnerabilities containing 34,164 CVE-IDs were included within the given time.

#### Exploits

Previous work used Symantec’s anti-virus attack signatures and Intrusion Detection Systems’ (IDS) attack signatures as class labels. The ground truth means that a vulnerability has been exploited in the wild and can represent the most real performance of the prediction model. It has been proved that Symantec’s signatures are the best available indicator for the vulnerabilities targeted in exploits kits available on the black market [10][12]. However, this ground truth cannot cover all the exploits in the wild due to the limitation of the actual scope of business of Symantec. Meanwhile, this ground truth is found to be biased towards reporting exploits targeting specific vulnerabilities in software products from certain vendors (e.g., Microsoft, Adobe) [11]. In this paper, we use the traditional exploit databases as a criterion to judge whether a vulnerability has been exploited or not while use Symantec antivirus attack signatures and Intrusion Detection Systems (IDS) attack signatures to judge whether a vulnerability has been exploited in the wild or not. Two data sources are used to indicate whether a vulnerability is exploited, namely exploits on Exploit-DB (EDB) and SecurityFocus. The Exploit Database is a CVE compliant archive of public exploits and corresponding vulnerable software maintained by Offensive Security. SecurityFocus maintains an exploit page including exploits collected from various blogs and security reports and determined by security experts, which indicates whether a vulnerability has a corresponding exploit. We did not collect data sources such as exploit kits, studied by Allodi et al. [9], considering that the process of data collection is very cumbersome and resulting models would not have been comparable with previous research. Reckoning for the narrow coverage of Symantec signatures, we use it as supplementary data to conduct a separate experiment to compare with previous work. Metasploit is the world’s most used open source penetration testing framework, which can be used to conduct effective penetration tests on remote computer systems [42]. Metasploit maintains a highly exploitable database of exploits [43], implying that the possibility of a vulnerability being exploited in the wild increases.

We used the presence of a sentence–“Currently the SecurityFocus staff are not aware of any exploits for this issue.”–in the SecurityFocus exploit page to choose whether to label a vulnerability as exploited or not. Then, we exclude invalid data with candidate number that was not associated with any vulnerability within a given time from NVD and regard whether there exists related PoC in EDB as ground truth label for vulnerabilities indexed by CVE-ID on NVD. MITRE maintains a reference map [44] for EXPLOIT-DB to associated CVE entries or candidates. However, the reference map has been proved to be incomplete [15]. We use a web scraper to determine whether there is an external CVE reference for each exploit on EDB. Finally, the number of vulnerabilities and the proportion of vulnerabilities exploited are shown in Table 1. Besides, for 10,847 CVEs appearing on both SecurityFocus and NVD, 1,281 vulnerabilities on NVD have PoC exploits, and the exploited vulnerability on SecurityFocus includes 1,280 of them.

**Table 1:** Number of vulnerabilities, number of exploited vulnerabilities, the fraction of exploited vulnerabilities that appeared in each source.

	SecurityFocus	NVD
Number of vulnerabilities	29,388	56,681
Number of exploited vulnerabilities	10,876	3,784
Percentage of exploited vulnerabilities	37.008%	6.676%

### Features description

In this section, representative and meaningful features are combined, including numerical, categorical, and binary features (see Table 2). These features are described in detail below.

**Table 2:** Summary of data features, types and modelling method.

Feature Source	Raw data		Category	Modelled Method
Open Source Vulnerability Data	CVSS	Access Vector	Categorical	One-hot Encoding
		Access Complexity	Categorical	One-hot Encoding
		Authentication	Categorical	One-hot Encoding
		Confidentiality Impact	Categorical	One-hot Encoding
		Integrity Impact	Categorical	One-hot Encoding
		Availability Impact	Categorical	One-hot Encoding
		Base Score	Numeric	Scaled to N(0,1)
		Exploitability Score	Numeric	Scaled to N(0,1)
		ImpactScore	Numeric	Scaled to N(0,1)
	CPE	Application	Binary	Binary Vector
		Hardware	Binary	Binary Vector
		OS	Binary	Binary Vector
		No Product	Binary	Binary Vector
		List	Text	fastText
	CWE	CWE-ID	Numeric	One-hot Encoding
	Product Count		Numeric	Scaled to N(0,1)
	OVAL		Binary	Binary Vector
	Description		Text	fastText
Exploit	Label		CVE-ID	1 ⇔ CVE-ID *in* PoC DB
Exploit in the Wild	Label		CVE-ID	1 ⇔ CVE-ID *in* Attack Signatures

#### CVSS

The correlation vector of CVSS is determined by NVD security experts, capturing the characteristics of a vulnerability that are constant with time and across user environments, which can be used to evaluate the severity of vulnerabilities and help determine the urgency and importance of the required response. We use version 2.0 CVSS base score as a feature for our classifier. This set of features includes the vulnerability assessment scores ranges from 0 to 10 and the correlation vectors that determine the scores. The assessment scores include CVSS base score, exploitability subscore and impact subscore. Exploitability subscore is calculated from the values assigned to the feature’s Access Vector, Access Complexity, Authentication while impact subscore from Confidentiality Impact, Integrity Impact, and Availability Impact. The base score is calculated from the values of all feature vectors mentioned above. The measures in the vector are predetermined from fixed values and can accurately describe a vulnerability from various aspects. Access Vector reflects how the vulnerability is exploited and can take on the values “requires local access”, “adjacent network accessible” or “network accessible”. Access Complexity indicates the complexity of the attack required to exploit the vulnerability once an attacker has gained access to the target system and can be rated “high”, “medium” or “low”. Authentication measures the number of times an attacker must authenticate to a target in order to exploit a vulnerability and can take on the values “requires multiple instances of authentication”, “requires single instance of authentication” or “requires no authentication”. Confidentiality Impact, Integrity Impact, and Availability Impact indicate the impact to system or product of a successfully exploited vulnerability and can be scored as “none”, “partial” or “complete”.

#### CWE&CPE

Common Platform Enumeration (CPE) is a standardized method of describing and identifying classes of applications, operating systems, and hardware devices present among an enterprise’s computing assets. Based on CPE URI, we extract the corresponding affected platforms and products from the vulnerabilities released by NVD. CWE is a community-developed list of common software security weaknesses. CWE can help fine-tune the determination of the categories of flaws that can be unintentionally made during software development and can exist in software architecture, design, or code.

#### OVAL

Open Vulnerability and Assessment Language (OVAL) definitions define exactly what should be done to verify a vulnerability or a missing patch. We chose the existence of an OVAL definition as a feature.

### Text feature

When a vulnerability appears online, there is always relevant text description about the vulnerability, which summarizes the defective system/software and even gives method on how to utilize the vulnerability. Based on the reiterative analysis of the experiments in the past literature, we found that the most important feature that affects the prediction results is vulnerability summary, which will be given a detailed description in Section 4. Therefore, we have focused our work on this feature.

In the past work, the TF-IDF model [45] was used to vectorize the text. TF-IDF creates a vocabulary of all the words in the description. The importance of a word increases in proportion to the number of times it appears in the document, but decreases in inverse proportion to the frequency of its occurrence in the corpus. This eliminates common words from being important features. However, this method may fail for rare words or spliced words as it mistakenly regards these words as features of description. For example, in the vulnerabilities disclosed in NVD from 2013 to 2018, “privilege-escalation” appeared only once while “privilege escalation” appeared 340 times. Besides, traditional text processing techniques represent statistical features of words, but fail to capture the semantic similarity between words.

**Fig 4:**
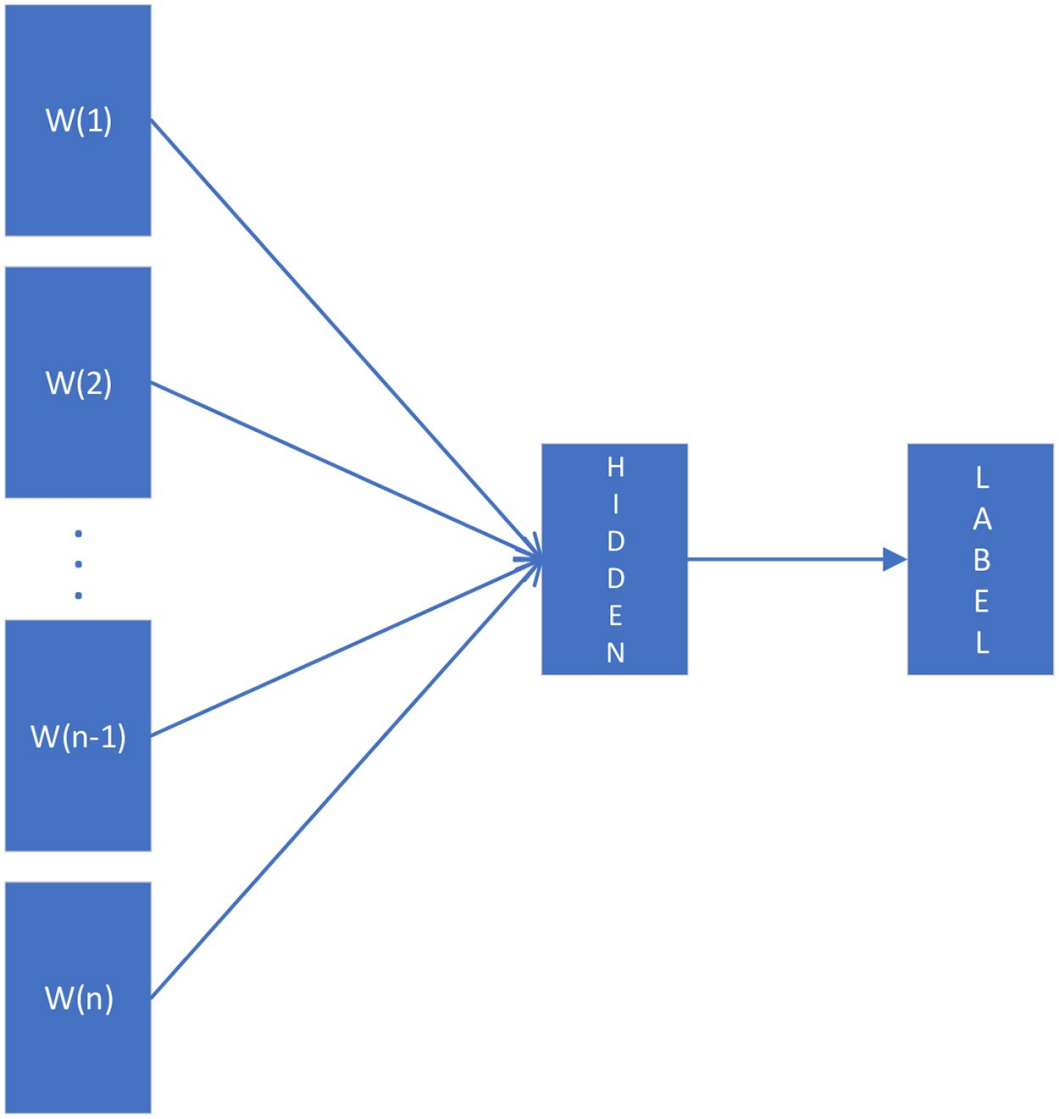
Model architecture of fastText classification model.

We select fastText proposed by Joulin et al. [23, 24] to train a neural language model, and its classification results or the sum of word representations will be combined with other features as input to the final prediction model. For short texts related to vulnerabilities, using fastText classification model can often achieve good results. For long vulnerability-related texts such as security blogs, using fastText word embedding model to embed text into segment vectors may achieve better results. For fastText word embedding model, fastText follows the inspiration of word2vec [46] to learn to embed words with similar semantics into similar vector space effectively. Besides, fastText combines bag of words and bag of character n-grams to represent a sentence. By considering the character n-grams, better word embeddings are generated for rare words. We use CBOW model with Negative Sampling to represent words in the paper. For fastText classification model, its model architecture is shown in Fig 4. *W*(1) to *W*(*n*) in Fig 4 represent the word embedding representation of each word in the document. Then vulnerability-related documents can be represented by the average of all word embeddings, which is shown in the following formula.

$$\begin{array}{c} h_{doc}=\frac{1}{n}\sum_{i=1}^{n}w_{i} \end{array}$$ (1)

The fastText classification model is similar in structure to the CBOW model in word2vec. The difference is that fastText classification model is supervised learning model, and its goal is to average all the word embeddings in the document and get the class label from the hidden layer through a non-linear transformation. FastText improves the performance of basic linear classifiers with fast loss approximation and the key features of rank constraint. The word n-gram feature can deal with the problem of missing word order in fastText model to some extent. Therefore, word n-gram embeddings are also regarded as word embeddings and are added to the hidden layer to be averaged with other word embeddings.

The optimization objective of the fastText classification model is to minimize the negative log-likelihood function as follows:

$$\begin{array}{c} -\frac{1}{N}\sum_{n=1}^{N}y_{n}\log(f(BAx_{n})) \end{array}$$ (2)

Among them, *x*_*n*_ is the normalized BoW of the n-th vulnerability description, *y*_*n*_ the prediction label. Matrix *A* is a word-based look-up table, which is the word embedding vector. *Ax*_*n*_ means to look up the representations of all the words in document and average them. Then the representation of the document is fed into the linear classifier, which corresponds to matrix *B* in the above formula. Finally, the probability distribution over pre-defined classes is obtained by using the softmax function *f*. The softmax function is shown below, which normalizes the value of the output layer of the fastText model to a range of 0-1.

$$\begin{array}{c} \mathrm{softmax}(z_{j})=\frac{e^{z_{j}}}{\sum_{k=1}^{K}e^{z_{k}}}\;\text{for}\;j=1,\ldots ,K \end{array}$$ (3)

Among them, *K* is the number of categories output by the fastText model. In our case, *K* equals 2. *z*_*j*_ equals *BAx*_*n*_, which is the output of the linear classifier mentioned above. So the output values of the softmax function are interrelated, and the sum of their probabilities is always 1.

FastText is much faster and can achieve performance equivalent to the methods based on deep learning. In particular, the descriptions of different vulnerabilities are variable-length, while fastText is more suitable for such data sets than some traditional machine learning methods or deep learning methods such as TextCNN [47].

In this work, we concatenate all texts with the data type Text in Table 2 together into one longer entry and implement lemmatization and removal of stop words on the text at first, and then we use fastText to classify the text or learning the embeddings of the text. The result of fastText’s classification or the embedding representation is one of the features of the final prediction model.

### Data labeling and classifier

The exploited vulnerabilities are labeled with related PoCs in exploit database while the vulnerabilities exploited in the wild labeled with authoritative attack signatures.

Random Forest (RF) is one of the most commonly used algorithms for classification and other tasks. However, the main limitation of random forests is that the use of a large number of trees will slow down the speed, resulting in a lack of real-time prediction capability. We choose the LightGBM algorithm [25] as the exploit predictor, which is a fast, distributed, high-performance gradient boosting algorithm [48] based on decision tree and performs well on unbalanced data sets. LightGBM fastens the training procedure and achieves higher accuracy by using a histogram-based algorithm and generating more complex trees by following leaf-wise split approach [25]. The idea of histogram algorithm is to convert continuous floating-point eigenvalues into discrete values (*k*) and construct a histogram with a width of *k*. Then the model traverses the training data and counts the cumulative statistics of each discrete value in the histogram. In feature selection, the best segmentation point can be found by traversing the discrete values of the histogram. LightGBM eliminates the level-wise decision tree growth strategy currently used by most GBDT and uses the leaf-wise strategy with depth limitations. The leaf-wise strategy is to find the leaf with the highest splitting gain from all the current leaves and then split each time. Therefore, compared with the level-wise strategy, the leaf-wise strategy can reduce more overhead and errors and obtain better accuracy under the same splitting times. Considering the over-fitting caused by leaf-wise strategy, LightGBM added a factor named maximum depth limit to the model. This parameter can prevent over-fitting and ensure high efficiency.

Grid search [49] across 10-fold cross-validation on the training set is used to determine the optimal hyper-parameters. Grid search sets a list of values for different hyper-parameters, and then traverses each hyper-parameter combination for performance evaluation to select the hyper-parameter combination with the best performance.

## Experimental evaluation

To evaluate our model, we conducted a series of experiments. All parameters of the methods are optimized to achieve the best performance. We performed the experiments using a server with an Intel(R) Core(TM) i7-7700 CPU @ 3.60GHz, 16G RAM, and NVIDIA GeForce GTX 1060 6GB. The scikit-learn Python package [50] was used.

### Performance evaluation

The problem of exploit prediction is essentially a two-class problem, that is, whether a vulnerability will be exploited or not. So the performance evaluation is done by using statistical measures of binary classification. They are computed as shown in Table 3 and details are described as follows:

**Table 3:** Classification performance metrics.

Metric	Formula
Accuracy	$\text{Accuracy}=\frac{TP+TN}{TP+TN+FP+FN}$
Precision	$\text{Precision}=\frac{TP}{TP+FP}$
TPR (recall in case of binary classification)	$\text{Recall}=\frac{TP}{TP+FN}$
F1	$F1=2\frac{\;\text{Precision}\;*\;\text{Recall}\;}{\;\text{Precision}\;+\;\text{Recall}\;}$
FPR	$\text{FPR}=\frac{FP}{FP+TN}$

P-true positives, FP-false positives, FN-false negatives, TN-true negatives.

Accuracy of exploit prediction is the correct classification of True Positive (TP) and True Negative (TN), where TP is the number of exploited vulnerabilities that are correctly identified, FN is the number of exploited vulnerabilities that are mistakenly classified as vulnerabilities that will not be exploited. Precision indicates the fraction of vulnerabilities that were exploited from all vulnerabilities predicted to be exploited by our model. It highlights the effect of misclassified non-exploited vulnerabilities. Recall indicates the fraction of correctly predicted exploited vulnerabilities from the total number of actually exploited vulnerabilities. It highlights the effect of misclassified exploited vulnerabilities and is the most critical indicator for our exploit prediction model. The higher the recall is, the smaller the loss caused by exploited vulnerabilities will be. F1-score is the harmonic mean of precision and recall and represents the result of a trade-off between precision and recall. Precision-recall curves can graphically show the results of this trade-off as recall changes. The Receiver Operating Characteristics (ROC) curve visualizes the performance of the classifier by plotting the true positive rate and false positive rate on various thresholds of the confidence score output by the classifier. The Area Under Curve (AUC) is defined as the area enclosed by the coordinate axis under the ROC curve. Classifiers with larger AUC have better performance.

### Base model vs. fastEmbed

The work of this paper is inspired by Bullough et al. [15], who uses TF-IDF with a maximum of 2000 terms to quantify text features and a support vector machine (SVM) with linear kernel as the classifier. Due to the difference in experimental data sets–newer vulnerability data is used in our work–we reproduced Bullough’s work. We got almost identical results on the same data set, which enabled the following experiments to be carried out. The same method was adopted as a basis for replication. Then the obtained model was used as the base model for all subsequent experiments. The features input to the models in all experiments are shown in Table 2 and are all used for comparison. In order to demonstrate the effectiveness of the model in real production environment, we did not adopt the method of balancing classes reported by Edkrantz [8] and Bozorgi [16].

In the experiment, we evaluated the performance of the proposed approach using 10-fold stratified cross-validation. In 10-fold stratified cross-validation, the dataset is divided into ten equal size subsets while ensuring that the proportion of all kinds of samples in the training set and test set is the same as that in the original data set. Nine subsets are retained as training data, and the remaining one subset is used as the validation data for testing the model. The cross-validation process was repeated ten times, and the final result was obtained from the average value of the results. In particular, for the fastEmbed model we proposed, we split the original data set at a ratio of 3:7, of which 30% is used for supervised learning to train a text classifier, and the remaining 70% is input into the LightGBM model for cross-validation in combination with the prediction results of fastText. In other words, we did 10-fold stratified cross-validation on the remaining 70% data. This method eliminates the risk that the fastText model is over-fitted to the training set and the training label is indirectly introduced into the training set, which is caused by directly carrying out 10-fold cross validation on the entire data set.

**Fig 5:**
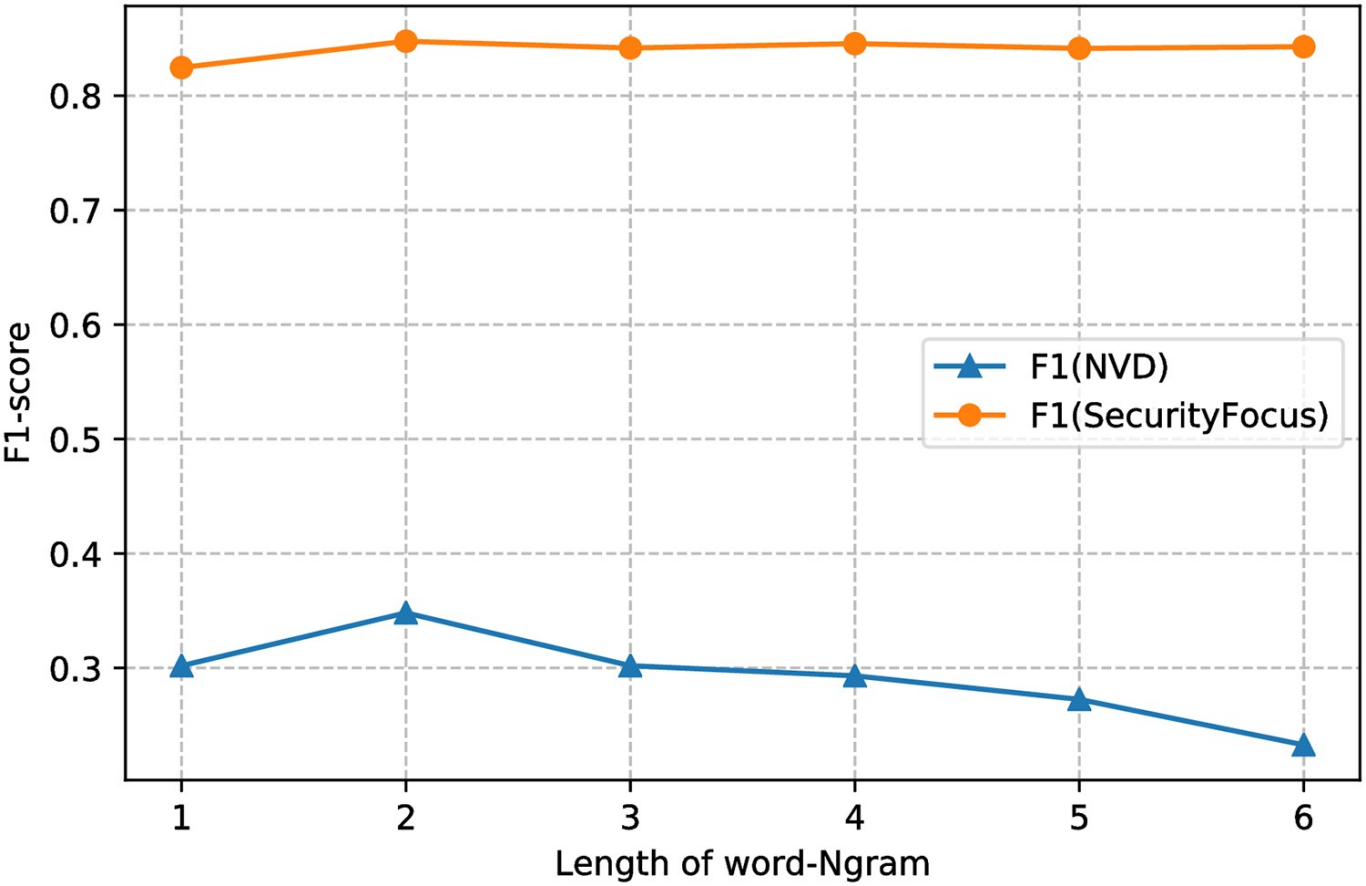
Evaluation by using different length of word N-gram.

First of all, how to make the model capture the details of vulnerability description to the greatest extent and learn continuous segmentation embedding to make up for the lack of segmentation and word order is vitally important. We choose different n-gram size to optimize the model by 10-fold stratified cross-validation. Fig 5 presents the F1 measure of the model with varying lengths of word n-grams. The results show that 2-gram makes the model achieve the best effect when considering the performance on SecurityFocus and NVD databases at the same time.

Then we optimized the parameters including loss function, the dimension of the embedding, the minimal number of word occurrences, learning rate and the size of the context window by grid search [49]. We adopt hierarchical softmax as the loss function, which is linear to logtime and improves the computational complexity of the softmax function. The higher dimension space can help to understand the vulnerability description; at the same time, it needs more space and brings the problem of sparse representations. The size of the context window is a quantitative indicator corresponding to the context a word contains. The larger the size of the context window, the more time the model will spend on training and the more accurate the context it can capture. The minimal number of word occurrences can filter the word frequency of the word list to avoid the word list being too large. Learning rate is a hyper-parameter that guides how to adjust the weight of neural network through the gradient of loss function. The lower the learning rate, the slower the change speed of the loss function and the more time it takes to train the model. Based on the above knowledge, the resulting parameters and ranges for the grid search can be seen in Table 4.

**Table 4:** Parameters used for our fastEmbed algorithm, and the range for hyper-parameter tuning using grid search.

	Embedding size	Occurrences	Learning rate	Window size
Parameter value	65	1	0.06	20
Range for tuning	[30, 160] by 5	[1, 10] by 1	[0.01,1] by 0.05	[5, 50] by 5

Embedding size-the size of word embedding, Occurrences-the minimal number of word occurrences, Learning rate-the learning rate of neural network model, Window size-the size of the context window.

Finally, we evaluated the base model of the combination of TF-IDF and SVM and fastEmbed with 2-gram. In order to prove the effectiveness of the text classifier used in the fastEmbed model, we replaced the text classifier in the fastEmbed model with a deep learning method called TextCNN [47] and conducted a comparative experiment with the fastEmbed model. TextCNN algorithm is one of the classic and effective methods in the field of text classification. We use word2vec [46] method to embed the words of vulnerability-related text into vector space of 65 dimensions, and then feed all the word vectors in vulnerability-related text into textCNN model for training and testing. Besides, the combination of paragraph vectors [21] and LightGBM has also been tested. Similarly, in order to prove the effectiveness of the exploit predictor, we chose LightGBM algorithm, SVM algorithm, random forest algorithm [51] and logistic regression algorithm [52] as classifiers to conduct cross-validation experiments respectively. Without the features of Table 2, we used the text classifier fastText alone to carry out cross-validation experiments to prove the effectiveness of the remaining features. The results of Precision, Recall, and F1 score derived from the models are shown in Table 5.

**Table 5:** Evaluating results.

	Model	Accuracy	Precision	Recall	F1-score
SecurityFocus	TF-IDF+SVM	0.846	0.797	0.775	0.781
	TF-IDF+LightGBM	0.859	0.819	0.779	0.793
	fastEmbed	**0.872**	**0.872**	**0.822**	**0.846**
	fastText	0.836	0.819	0.798	0.801
	paragraph vector+LightGBM	0.813	0.776	0.704	0.730
	CNN+LightGBM	0.865	0.872	0.802	0.802
	fastText+random forests	0.871	0.856	0.807	0.832
	fastText+SVM	0.871	0.869	0.822	0.845
	fastText+logistic regression	0.871	0.868	0.8215	0.844
NVD	TF-IDF+SVM	0.924	0.328	0.115	0.161
	TF-IDF+LightGBM	0.927	0.351	0.109	0.153
	fastEmbed	**0.933**	**0.621**	**0.251**	**0.356**
	fastText	0.921	0.411	0.205	0.257
	paragraph vector+LightGBM	0.926	0.391	0.034	0.055
	CNN+LightGBM	0.929	0.567	0.173	0.264
	fastText+random forests	0.920	0.430	0.251	0.311
	fastText+SVM	0.931	0.589	0.196	0.293
	fastText+logistic regression	0.929	0.554	0.187	0.279

**Fig 6:**
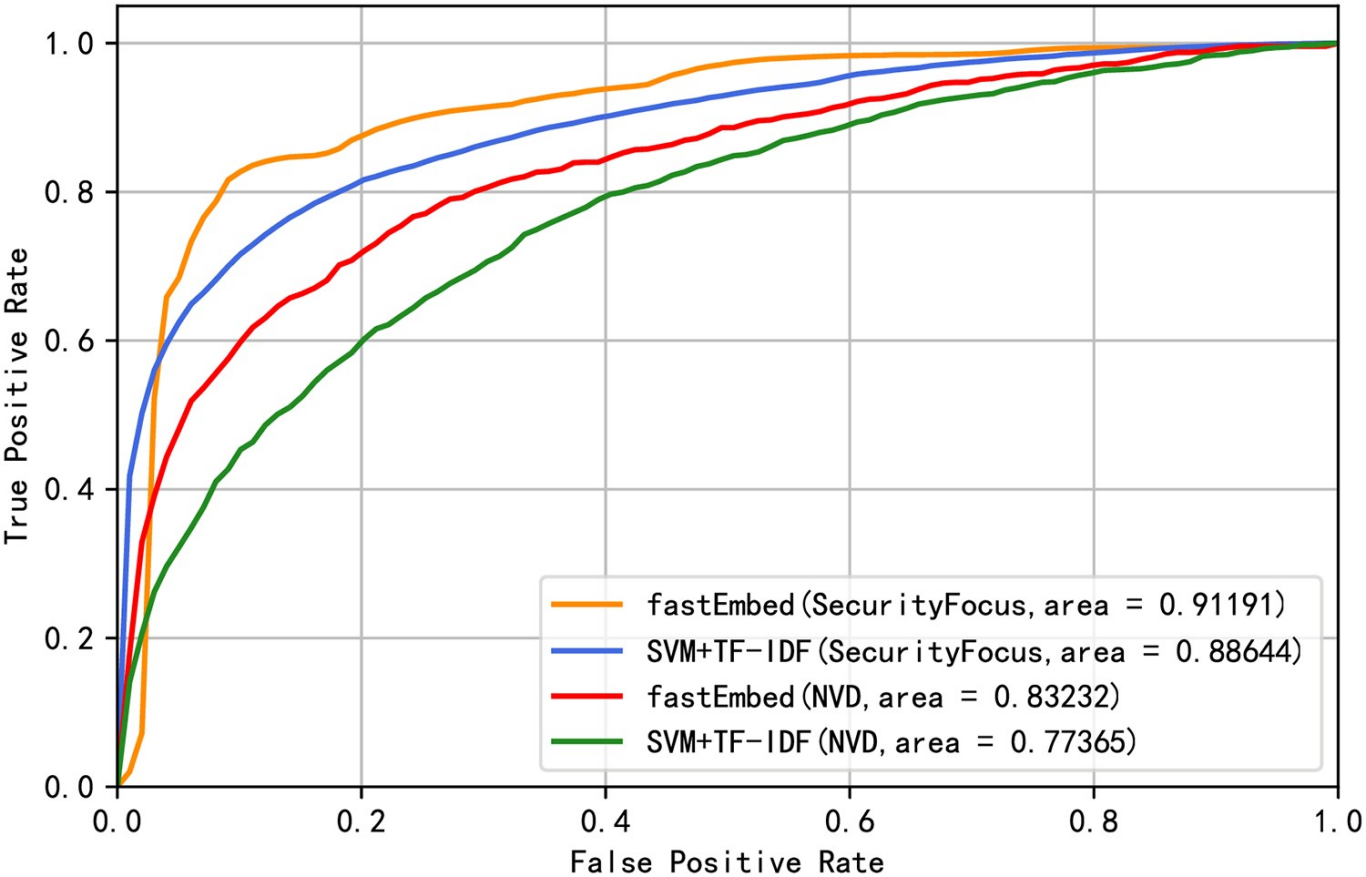
ROC curve of baseline model and fastEmbed.

**Fig 7:**
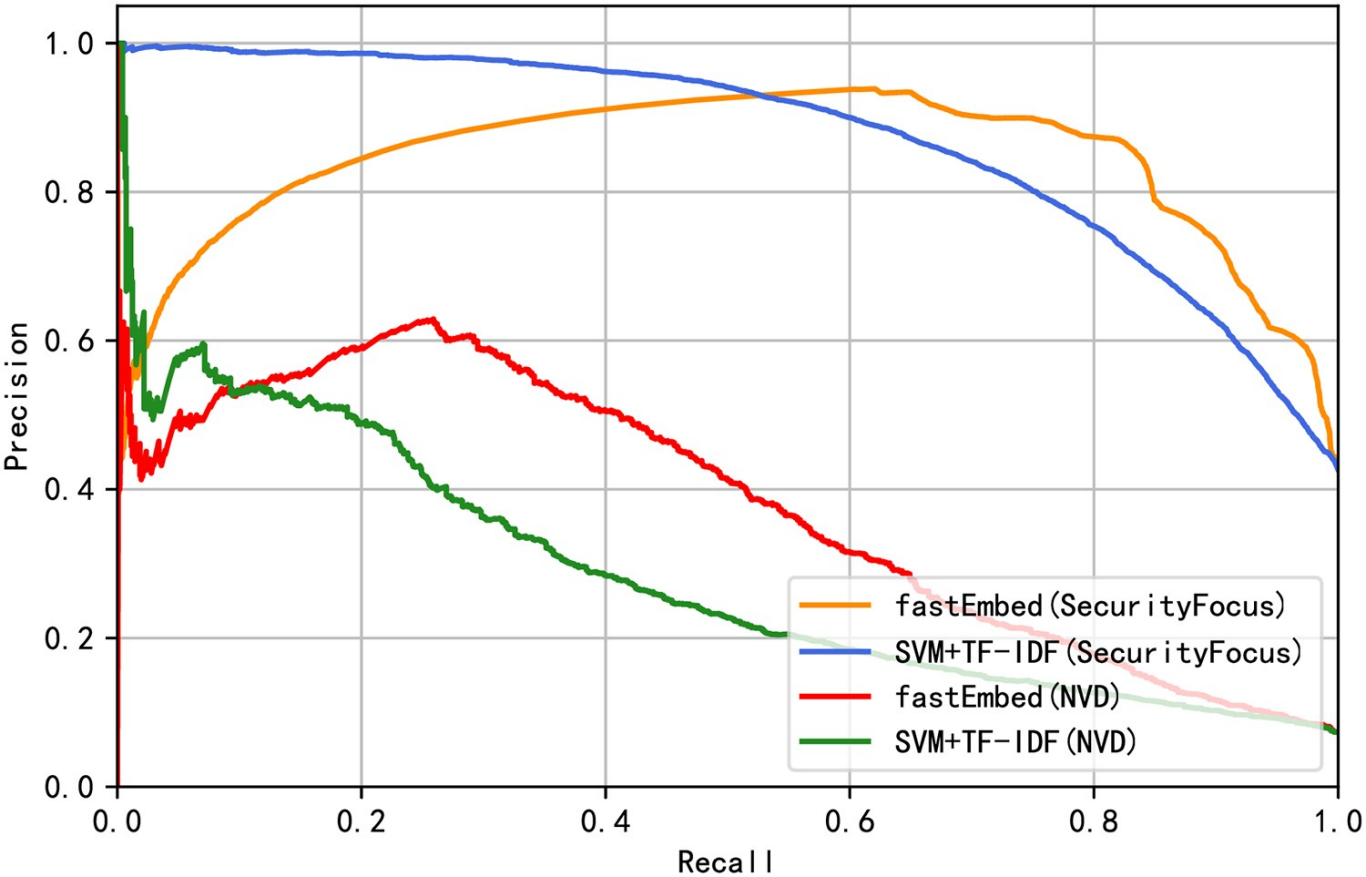
Precision-Recall curve of baseline model and fastEmbed.

Table 5 shows that the performance of the fastEmbed model is better than the baseline model. At the same time, it can be seen from Table 5 that the fastEmbed model is more accurate in identifying vulnerability-related texts under the condition that the exploit predictor is not changed. Under the condition of keeping the text classifier unchanged, fastEmbed model is more effective in exploit prediction. From the results in Table 5, it can also be inferred that the text classifier fastText contributes more to the proposed method. The reason why this happens is that text feature is the most important feature affects the final vulnerability exploit prediction results, which will be proved in the subsection of Feature Importance. Judging from F1 value, fastEmbed model outperforms other models above in overall performance. To demonstrate the prediction performance of our proposed method, a receiver operating characteristic (ROC) curve calculated by the fastEmbed model and base model is plotted as shown in Fig 6. We earned a better ROC curve with the area under curve (AUC) value of 0.91. Since the data are unbalanced, we continue to evaluate the models using Precision-Recall curves. The Precision-Recall curves of the models are shown in Fig 7. The result shows that performance was improved by applying our model. Besides, in order to prove that the result improvements from the base model and fastEmbed model are statistically significant, we conducted a t-test significance test [53] on the experimental results of 10-fold cross-validation. It is assumed that null hypothesis has no significant difference between the two groups of data, while alternative hypothesis has significant difference. The p-value calculated on the SecurityFocus and NVD data sets are 0.0058 and 0.0050 respectively. When the value of the significance level is 0.05, we reject null hypothesis and accept alternative hypothesis, which means that the improvement of fastEmbed model results is statistically significant.

When we use fastEmbed to evaluate the data from 2009 to 2015 on NVD, the average F1-score of the model can reach 0.6839. The fastEmbed model still outperformed the base model, which reached 0.647 in F1-score. When the model is applied to the data from 2013 to 2018, the performance of the model on SecurityFocus does not show large fluctuations. However, the effect of the model on NVD data shows an apparent decline. The reason for this result may be attributed to the following points. From the perspective of data labeling, we label NVD data with exploits from Exploit Database. However, the class labeling of NVD dataset is not sufficient, which means that some vulnerabilities existing related PoCs have not been labeled. Besides, the enthusiasm of security researchers for submitting exploits to Exploit-DB decreases. 16.78% of the vulnerabilities were exploited from 2009 to 2015, while only 6.68% from 2013 to 2018. On the other hand, although the first eight days is enough to make predictive models for exploitation [17], there may be some exploited vulnerabilities has no related exploit. Further, the data in Exploit-DB exists a certain tendency to publish CVE-related PoCs despite its relative high coverage rate of vendors, which will be proved in the subsection of Online Mode. When our model is applied to specific vendors in NVD and other class labeling sources are added, the performance of the model is further improved. From the perspective of vulnerability data, the explosive growth in the number of vulnerabilities has resulted in extreme imbalance in vulnerability categories and interfered with the judgment ability of the model. Besides, we will prove that vulnerability-related texts make most contributions to the performance of the model in the subsection of Feature Importance. However, many vulnerability descriptions on NVD are uploaded by individuals, and there may be ambiguity in the language description of NVD vulnerabilities.

### No temporal intermixing

For the prediction model, the method of dividing the training set and the test set randomly can be used as a benchmark to measure the generalization ability of the model. However, due to the concept drift phenomenon [38, 39], we need to prevent future information from leaking into the current working model as much as possible. This means that we arrange the data sets in chronological order, taking a certain time point as the boundary, using the data before the time point as the training data of the model and the data after the time point as the test data. The prediction model will predict future exploits based on past experience, thus avoiding future information leakage. For example, when a new attack mode appears, the past model may not understand the name or even the description details of this new attack mode. Therefore, the method of dividing the training set and the test set based on time series can reflect the most real predictive performance of the model. Different from the data partitioning method used in the experiment in the previous section, we use the method of training both fastText and LightGBM model on the training set to make the fastText model learn more about vulnerability description. At the same time, we divided the training set and test set in a specific proportion to compare with the previous work, of which the test set accounted for 18%.

**Fig 8:**
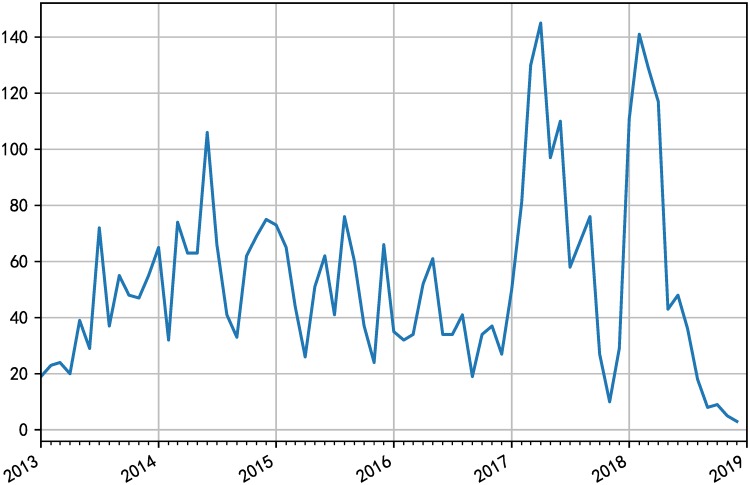
Exploit disclosures added to the Exploit Database (EDB) by month.

**Fig 9:**
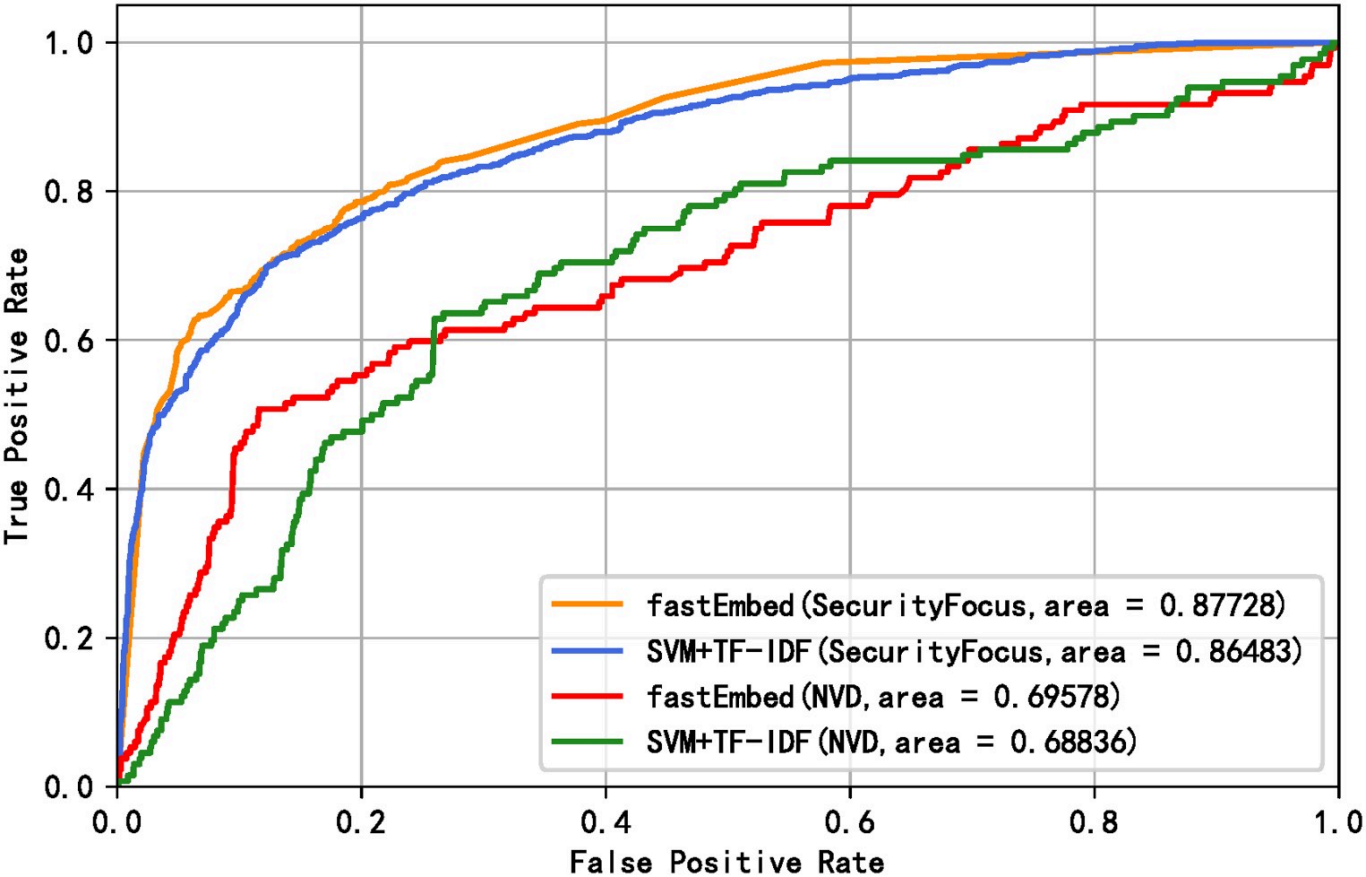
ROC curve of baseline model and fastEmbed without temporal intermixing.

**Fig 10:**
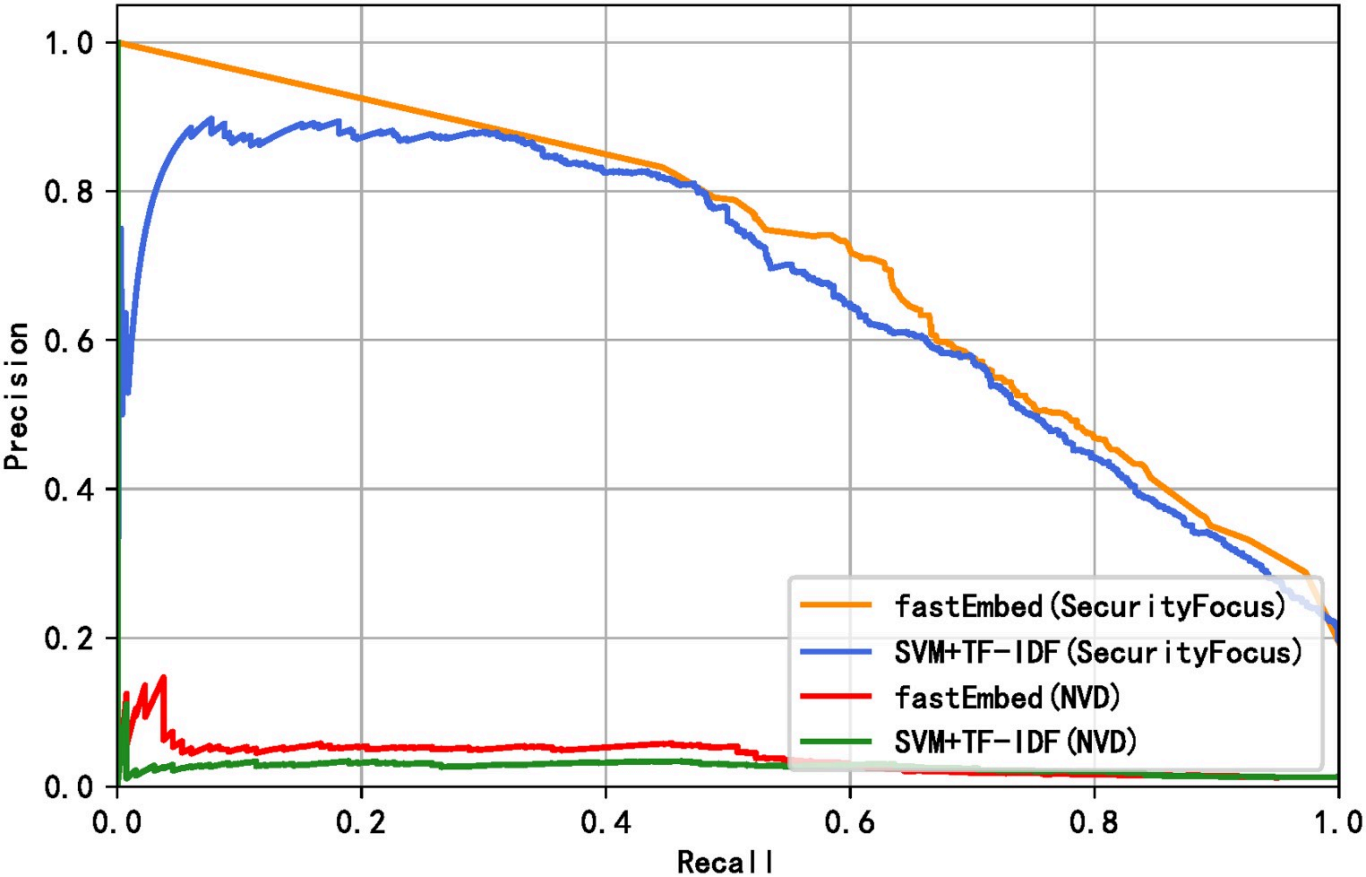
Precision-Recall curve of baseline model and fastEmbed without temporal intermixing.

Compared with the data released between 2009 and 2015, the imbalance of the data set used in this experiment has increased dramatically. Only 6.68% of the total number of vulnerabilities on NVD is exploited, while only 1.29% in the test set is exploited if the vulnerability data of NVD is divided temporally. Fig 8 shows the distribution of the number of exploits decreases over each month. We sort all the vulnerabilities by time and evaluate the models. Also, we verified the excellence of our model for the data from 2013 to 2018 and 2013 to 2017 respectively due to the particularity of temporal distribution of 2018. The results of Precision, Recall, and F1-score derived from the two models are shown in Table 6. Likewise, we draw the ROC curves and Precision-Recall curves corresponding to the model results, as shown in Figs 9 and 10 respectively. The evaluation results show that our model outperforms the basic model by 3.6% on average in overall performance and achieves better prediction ability with AUC of 87.7% on SecurityFocus.

**Table 6:** Performance comparison of baseline model and fastEmbed of splitting training and test sets temporally.

	SecurityFocus		NVD	
Model	TF-IDF+SVM	fastEmbed	TF-IDF+SVM	fastEmbed
Accuracy	0.824 (0.811)	0.857 (0.807)	0.984 (0.950)	0.973 (0.946)
Precision	0.535 (0.569)	0.622 (0.555)	0.030 (0.194)	0.054 (0.213)
Recall	0.710 (0.612)	0.674 (0.652)	0.008 (0.120)	0.068 (0.182)
F1	0.610 (0.590)	**0.647 (0.599)**	0.012 (0.148)	**0.060 (0.196)**

The data in brackets are the evaluation results from 2013 to 2017, while the data outside brackets are from 2013 to 2018.

**Fig 11:**
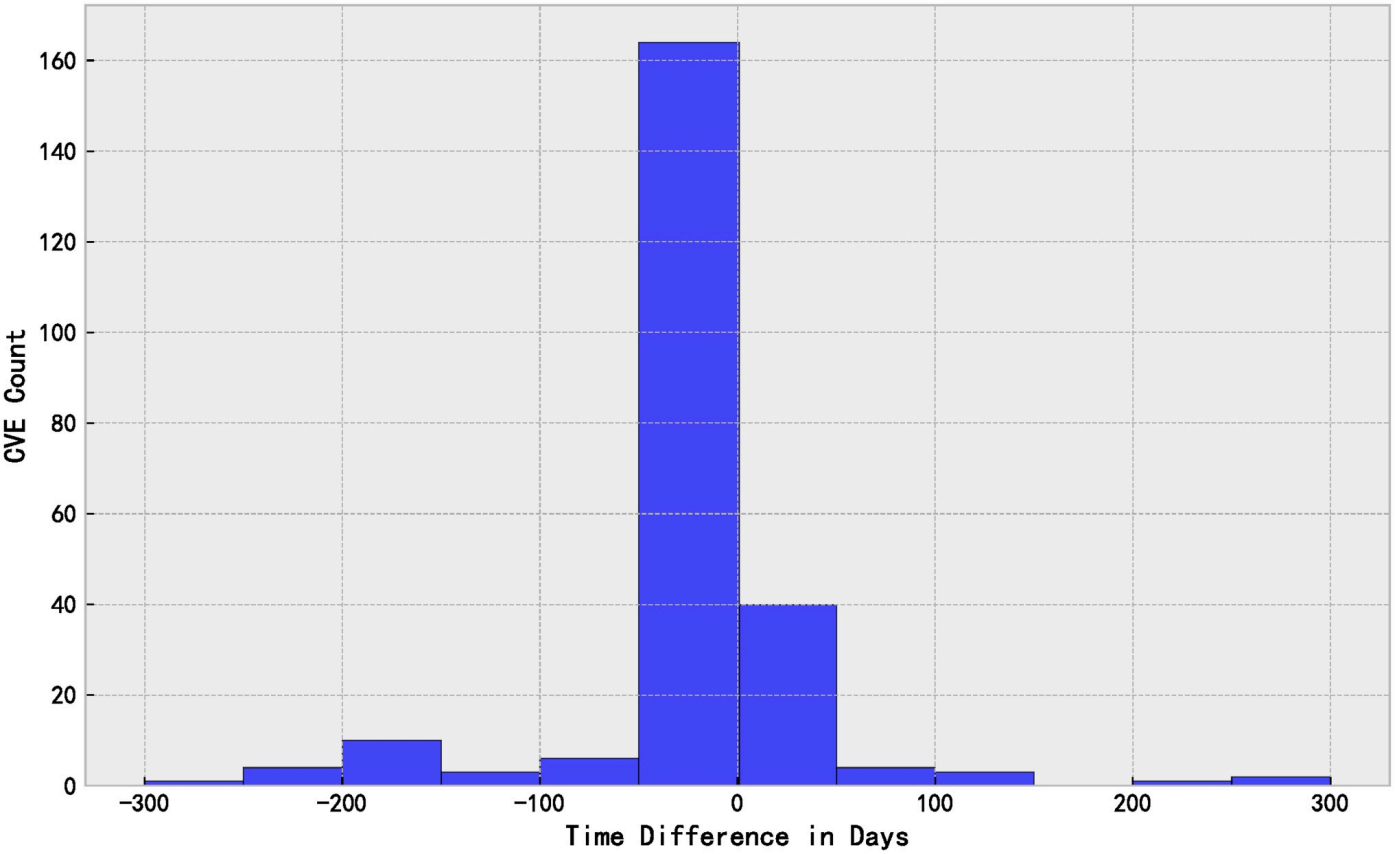
Distribution of the difference in days between vulnerability disclosure and exploit publication.

The performance on time of fastEmbed model on SecurityFocus is also verified. We collected PoCs from 12 mainstream exploit databases such as Metasploit, 0day.today [54] to determine the earliest release time of PoC since SecurityFocus does not provide time indicator of PoC. For “future” vulnerabilities divided according to time sequence, our model predicted 1957 vulnerabilities correctly out of 2710 exploited vulnerabilities when the ratio of training set to test set is 1:1, among which we determined 261 vulnerabilities with PoC release time. Fig 11 shows a histogram of the difference in days between vulnerability disclosure in the SecurityFocus database and exploit publication. 195 of 261 correctly predicted vulnerabilities are of practical significance, which means fastEmbed can predict the development of PoC in advance. Overall, our model can predict the release of PoC a median of 1 day ahead for these 261 vulnerabilities.

### Error analysis

We analyzed the prediction results of the fastEmbed model and base model on SecurityFocus in the experiment of No Temporal Intermixing subsection. The specific composition of the prediction results is shown in the Table 7. The results show that fastEmbed is slightly lower than the base model in the coverage rate of correctly predicted results, but it will reduce more unnecessary vulnerability repair investment. Furthermore, the fastEmbed model and the base model are 89.448% identical in prediction results. For different results predicted by the two models, the base model predicted 198 vulnerabilities correctly, while the fastEmbed model predicted 360 vulnerabilities correctly. After analyzing the wrong output results of the two models, we found that it is easier for the base model to statistically classify the vulnerability description containing phrases such as “code execution” and “bypass” as exploited. For example, 97 vulnerability descriptions incorrectly predicted by the basic model contain “code execution”, which is 25 in the fastEmbed model. At the same time, the base model is easier to predict incorrectly for some vulnerabilities with greater harm. For example, the description text related to the vulnerability numbered CVE-2017-8628 is “Microsoft Windows is prone to a security vulnerability that may allow attackers to conduct spoofing attacks. An attacker can exploit this issue to conduct spoofing attacks and perform unauthorized actions; other attacks are also possible.”. This vulnerability has certain harmfulness because of the widespread use of windows operating systems. The base model predicted incorrectly, but the fastEmbed model predicted correctly.

**Table 7:** Data composition of the test set and prediction results of the models.

	fastEmbed		TF-IDF+SVM	
	Negative sample	Positive sample	Negative sample	Positive sample
Test data set	4263	1025	4263	1025
The prediction results	4125	**1163**	3926	1362
Correctly predicted results	3807	707	3629	**728**

The real outputs of the fastEmbed model are also investigated, and the sampling results are shown in Table 8. For vulnerability with Bugtraq ID of 104595, its harmfulness is great due to the high coverage of products affected by the vulnerability. However, the exploitation value of the Denial of Service vulnerability with Bugtraq ID of 106031 may be relatively low. The fastEmbed model correctly predicts that these two vulnerabilities are exploited and non-exploited respectively. For the wrong outputs of the fastEmbed model, part of the reason is due to the wrong classifications or imperfect features of the fastEmbed model, and the other part of the reason includes incomplete labeling of the data set. For example, fastEmbed determines that the Microsoft Windows kernel information disclosure vulnerability with Bugtraq ID 106095 is exploited according to the semantics of the context related to the vulnerability. In fact, although the vulnerability has the value of exploitation, it may be difficult to exploit. Besides, the fastEmbed model incorrectly predicts the vulnerability with Bugtraq ID 103117, which has a high-risk impact on Drupal. Since Drupal is widely used to build websites around the world, there are already exploit codes for it online. However, the vulnerability is not exploited in our data set, although the fastEmbed model predicts it is exploited.

**Table 8:** Sampling prediction results of fastEmbed model.

Bugtraq ID	The prediction results	Class labels in data sets
104595	1	1
106031	0	0
103117	1	0
106095	1	0

### Online mode

We attribute the unsatisfactory performance of the model on NVD to the extreme imbalance of data categories and the rapid change of data distribution over time as shown in Fig 8, so we adopt the method of online prediction with class balancing on training data. The online mode method means that inputting the data of the previous year to the model for training, evaluating the data of the following year, and then adding the test data to the training data to continue training the model to evaluate the data of the following year. Much research has been done in the field of solving class imbalance [55]. Different sampling methods will achieve different results for different data sets and high-quality external threat intelligence sources. For vulnerability exploit data sets, Smote sampling algorithm performs better than oversampling for minority classes and undersampling for majority classes. So we use borderline Synthetic mind over sampling technique (Smote) [56] to balance the proportion of categories in the training set while the original ratio on the test set remains unchanged to ensure that the results reflect the effectiveness of the model in the real environment. This method focuses on some minority class samples of exploited vulnerabilities at the boundary of the optimal decision function and then generates samples in the opposite direction of the nearest neighbor class. In other words, Smote algorithm calculates the k neighbors of each minority sample, randomly selects n samples from the k neighbors for random linear interpolation, constructs a new minority sample, and combines the new sample with the original data to generate a new training set. We adjusted the sampling rate of the data in each year to make the sample ratio of the training set remain at 1:1. In particular, we set the ratio of positive samples to negative samples as 1:4 for the prediction of 2018 due to the extreme imbalance of data in time distribution as mentioned above. The same settings for rebalancing the data are used for both fastEmbed and base model. Table 9 reports the prediction results of different years using models based on the knowledge of the past few years. Based on the comparison of the evaluation results of each year above, it is further demonstrated that our model is slightly inferior to the basic model in the measure of recall but superior to the basic model in overall performance (F1-score) by 6.283%. The proportion of exploited vulnerabilities has decreased year by year, reaching only 3.700% in 2018. With the explosive growth of NVD vulnerability data and the increase of low coverage rate of exploit database, the performance of the model decreases year by year. There is an urgent need for high coverage of exploit data sets. When exploits in SecurityFocus are added as a label of whether the vulnerability is exploited, the performance of our proposed model is improved with F1 measure of 0.26, precision 0.18 and recall 0.42. When exploits from the 12 exploit databases mentioned above are added, the performance of fastEmbed is further improved with F1 measure of 0.38, precision 0.28 and recall 0.61.

**Table 9:** Performance metrics for prediction by year using online mode.

	SVM+TF-IDF			fastEmbed		
Year	Precision	Recall	F1	Precision	Recall	F1
2014	0.311	0.626	0.416	0.372	0.625	**0.466**
2015	0.256	0.535	0.346	0.306	0.444	**0.362**
2016	0.149	0.406	0.217	0.177	0.294	**0.221**
2017	0.108	0.338	0.164	0.144	0.255	**0.184**
2018	0.087	0.313	0.137	0.105	0.227	**0.143**

We investigate the exploited vulnerabilities each year, and the result shows that the data in Exploit-DB exists a certain tendency to publish CVE-related PoCs despite its relative high coverage rate of vendors. For example, Microsoft, IBM, Apple and Adobe are the four vendors that keep relatively stable in the number of PoCs published each year. In 2018, 40.72% of PoCs related to them were released, and the remaining vendors’ products accounted for less than 2% of PoC releases. We conducted separate experiments on vulnerabilities related to each vendor according to the above methods, taking the vulnerabilities in 2018 as test sets. The results of the experiment are shown in the Table 10, which proves the imbalance of PoC distribution among different vendors and shows that the performance of fastEmbed is obviously improved by predicting exploits of different vendors respectively and further improved by adding exploits from SecurityFocus, especially for Adobe.

**Table 10:** Performance metrics for prediction of different vendors by year using sliding window.

Vendor	Microsoft	Apple	IBM	Adobe
F1	0.287	0.260	0.269	0.400
F1 (Adding exploits from SecurityFocus)	0.302	0.290	0.419	0.619

### Feature importance

**Fig 12:**
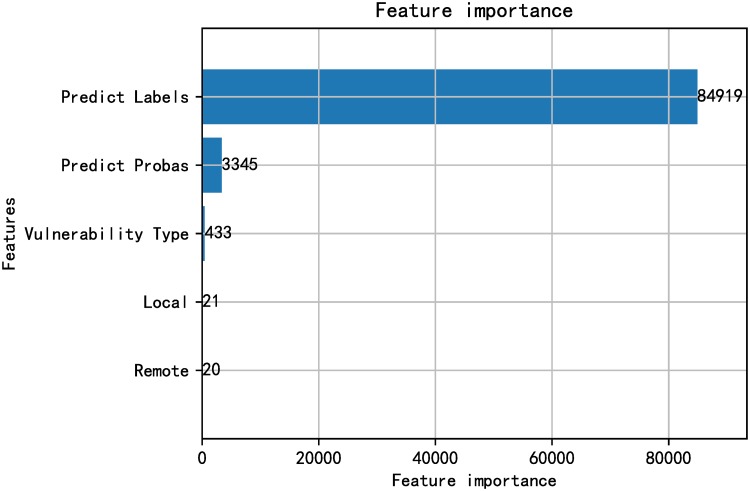
Feature importance ranking when performing fastEmbed model on SecurityFocus.

**Fig 13:**
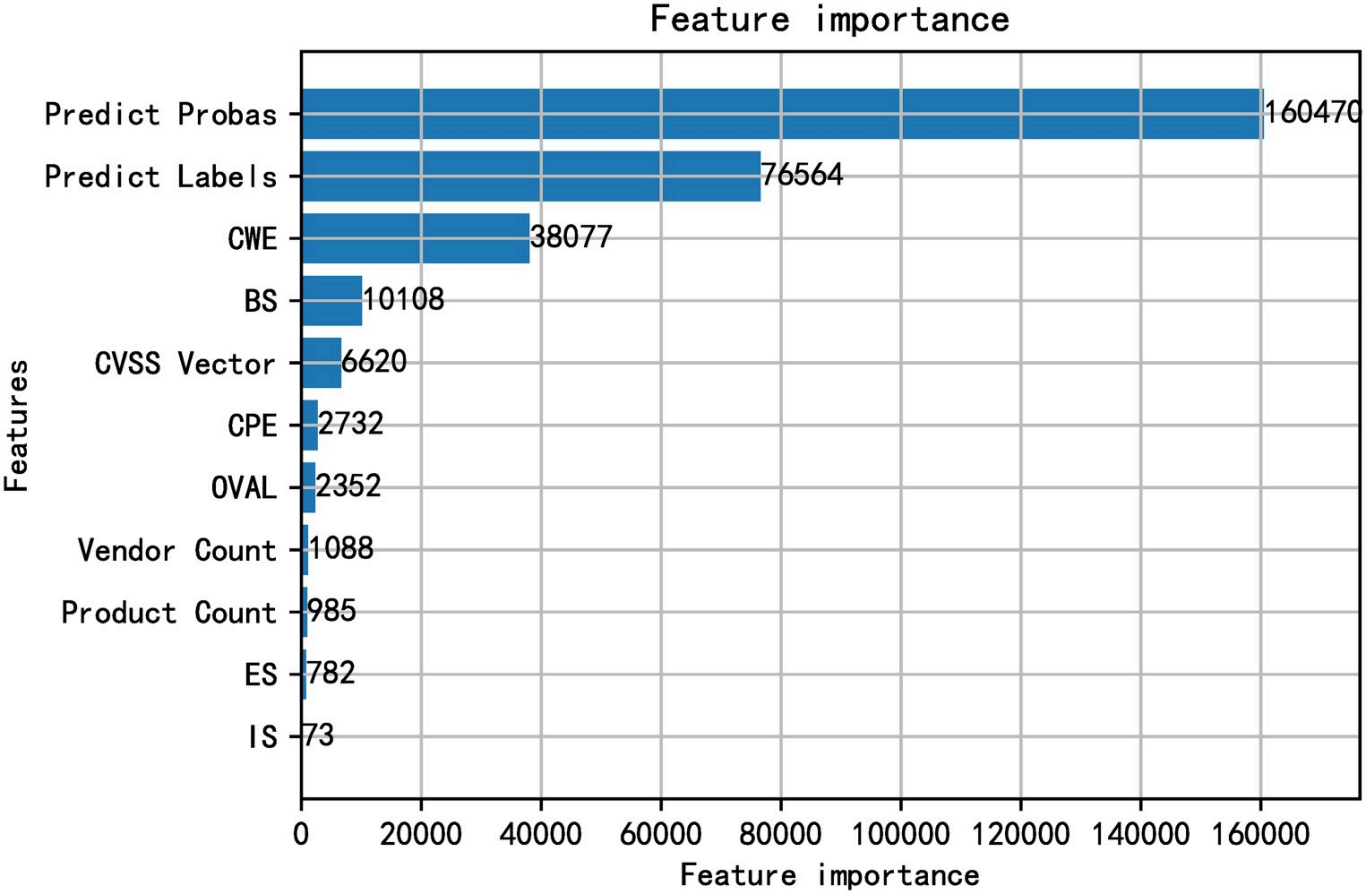
Feature importance ranking when performing fastEmbed model on NVD.

In order to better explain the validity of the features used in the model and the effect of the features on the decision-making of the model, several features that make the greatest contribution to the model are reported. The LightGBM classifier constructs some decision trees connected in sequence according to actual data. Similar to the Mutual Information (MI) [57] mentioned in [33], the total gain of splits which use the feature is calculated for each feature and describes the inherent relationship between adding features and how much the model performance will changes. All features used in our model are arranged in order of feature importance as shown in Figs 12 and 13. Unsurprisingly, the classification results and prediction probability values of vulnerability-related texts based on fastText have made most contributions to the performance of the model.

### Real-world exploits prediction

The data from Symantec anti-virus attack signatures and Intrusion Detection Systems(IDS) attack signatures are tested as exploits in the wild in order to corroborate the generalization ability of the model performance and provide a much-needed prediction capability.

First of all, we extracted data from the crawled signatures between 2015 and 2016, among them 124 IDS attack signatures have been reported without and 358 anti-virus attack signatures with their discovery date. In [11][33], experiments were conducted on vulnerabilities in Microsoft and Adobe products because Symantec did not have attack signatures for all platforms but provided the best coverage for vulnerabilities in Microsoft and Adobe products [11]. The same method is adopted. Therefore, 2,031 vulnerabilities targeting Microsoft and Adobe products were left, 358 of which were exploited in the wild regardless of their publishment time. For comparison, we evaluated the model on NVD. Based on the features shown in Table 2, whether there exists a PoC in Exploit Database or Metasploit Exploit Database is added as a binary feature and whether there exists a Symantec signature as a class label.

Next, we divide vulnerabilities according to a ratio of 3:7 as in the preliminaries section. Three-tenths of the data provides a training corpus for the fastText model while preventing the model from over-fitting data to other parts of data. Then 10-fold stratified cross-validation was performed on seven-tenths of the vulnerabilities.

**Fig 14:**
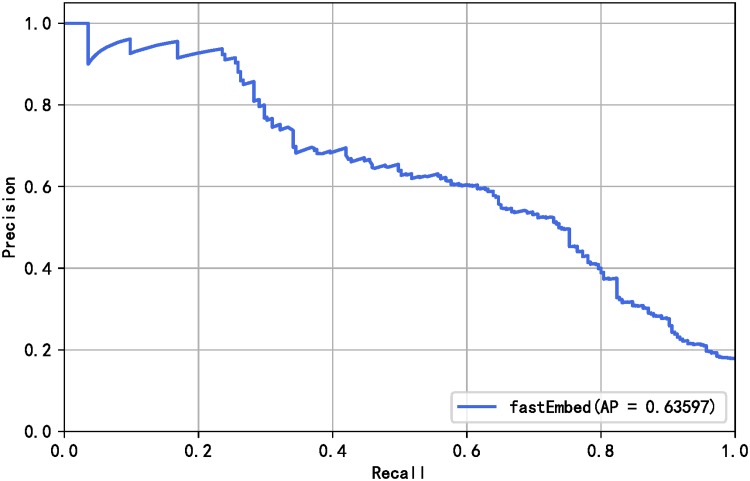
Precision-recall curve for Microsoft-Adobe vulnerabilities based on fastEmbed.

The prediction result of the model is promising, with F1-score reaching 0.586, recall 0.550 and precision 0.639. These measures are better than the results with F1-score of 0.44 achieved by [33]. The reported precision-recall curve is depicted in Fig 14 for comparison since the F1 measure is not reported in [11]. Also, the model was tested on data in 2018 and reached F1 measure of 0.601, recall of 0.560 and precision of 0.670. In short, our model achieves better performance without introducing features from social networks or darkweb/deepweb.

### Security blogs

There exists a gap in the ground truth of exploits in the wild [21]. In order to achieve higher coverage of ground truth, we crawled 7,956 blogs from 36 well-known security experts or security vendors from 2014 to 2018. Through regular expression matching CVE-IDs in posts, 536 blogs were left, 287 of which mentioned vulnerabilities exploited in the wild.

Since the aim is to identify blogs that describe exploited vulnerabilities, we use the fastEmbed model to implement 5-fold cross-validation on limited data. The fastEmbed model learns the embeddings of character n-grams and represents every word as a sum of the n-gram vectors [24]. Then we average all the word vectors in a blog as the feature of LightGBM classifier. For comparison with the past work, the SVM classifier on TF-IDF vectors and paragraph vectors [21] as feature respectively are also introduced. In this experiment, TF-IDF model uses a maximum of 3000 terms to construct vectors while Paragraph Vector model uses an embedding of size 150 since more feature dimensions do not bring more benefits. In order to prove the effectiveness of fastText embedding model on security blogs, we have introduced other embedding models word2vec, GloVe [58] and BERT [59] for comparison. The embedding dimensions of word2vec and GloVe are consistent with fastText at 150, and the pre-trained BERT model is used to embed the document. Also, CVSS score is used as a feature other than the document embedding vector. Table 11 shows the comparison of the performance of the models. The result shows that fastEmbed has better generalization ability and the potential to be applied to predict various threat intelligence-related contexts.

**Table 11:** Classification results on different methods.

Method	Accuracy	Precision	Recall	F1
TF-IDF+SVM	0.814	**0.856**	0.804	0.827
word2vec+SVM	0.812	0.820	0.856	0.835
fastText+SVM	0.828	0.840	**0.859**	0.847
DarkEmbed	0.789	0.806	0.815	0.810
fastEmbed	**0.830**	0.841	0.819	**0.852**
word2vec+LightGBM	0.816	0.838	0.841	0.837
BERT+LightGBM	0.770	0.781	0.819	0.797
GloVe+LightGBM	0.793	0.809	0.830	0.819

## Conclusion

In this paper, we propose a model to predict the exploitability and exploitation in the wild of vulnerabilities on extremely unbalanced data sets by grasping the key features of the vulnerability-related text, which is confirmed to be the most important feature of the model. Through our empirical research on vulnerability exploitability, the results show that with the explosive growth of vulnerabilities, PoCs existing in Exploit-DB shows obvious tendentiousness and incompleteness, resulting in a decline in overall performance on NVD–except for some vendors. On the contrary, some vulnerability intelligence with high coverage and performance advantages over time, such as SecurityFocus, perform well even under real environment. Besides, our experiments on exploits in the wild show that our model is promising on limited ground truth. Based on these insights, our model outperforms the existing methods and can predict the exploitability and exploitation in the wild of a vulnerability on SecurityFocus a median of 5 days ahead of existing data sets while ensuring high coverage of vulnerability data and robustness against adversaries. Moreover, our model predicts the release of PoCs a median of 1 day ahead from the perspective of the prediction results of the model and can be applied to various threat intelligence such as security blogs.

Further research is needed to improve the accuracy and quality of exploits labeling of exploit database and the proof of exploits in the wild. More data sources with high coverage and time efficiency should be investigated because of the promising results produced on SecurityFocus in this paper. Another direction is to apply this method to the posts of community groups that are more attractive and meaningful to hackers and provides early warnings for vulnerability exploitation such as darkweb/deepweb.
